# Case of Successful Extirpation of the Ovarium

**Published:** 1848-09

**Authors:** H. Miller

**Affiliations:** Louisville, Ky.


### RECORD OF MEDICAL SCIENCE.

*Case of successful Extirpation of the Ovarium.* By H. Miller, M. D., of Louisville, Ky.—Mrs. McLaughlin, aged 37 years, came to this city last winter, from the State of Indiana, for the purpose of consulting me in relation to a tumour in the abdomen, which was a source of no little annoyance to her on account of its size. On inquiry, I learned that she had first observed a tumour, of no great size, in the right iliac region, some time during the previous fall, (1837,) which increased rapidly till it acquired its present magnitude. The menses had only appeared once or twice, and that in small quantity and of short continuance, since the tumour first attracted her attention. She had felt no acute pain in the tumour, at any time, and her general health did not appear to have suffered much, her appetite and digestion being pretty good. On examination I discovered a large tumour in the abdominal cavity, extending considerably above the umbilicus, and mostly occupying the right side, but stretching across the linea alba. Its shape was irregularly globular, and its consistency variable, being, at some points, much firmer than at others,—and in portions of it, fluctuation could be distinguished. Pressure upon it, even rude handling, gave no pain, and it appeared to be, in some degree, moveable in the cavity.

Examined *per vaginam*, the tumour could be felt within the pelvis, and the uterus was found in its normal condition.

The history of the case, in connection with the examination I made, left no doubt on my mind, that the patient was laboring under enlargement of the right ovary, most probably caused by multilocular degeneration of the organ: but I had the advice of my friend and colleague, Professor Gross, who, after examination, concurred with me in opinion.

The patient was made acquainted with the nature of her disease, and informed that but little hope could be entertained of relief from medicine; that the only certain method of cure would be to remove the tumour with the knife, but that the operation might itself prove fatal. Notwithstanding the little encouragement given her, she was anxious for an operation, preferring the risk of death to her uncomfortable situation, and the prospect before her, should she be abandoned to her fate. I could not, under the circumstances, refuse to operate; but advised her, as the weather was cold, and her means of procuring comfortable accommodation scanty, (she is poor and a widow besides,) to return home and come back in the spring, when the weather would be more favourable. Some medicine was prescribed for her and a suitable regimen enjoined.

I hoped the poor woman might be benefitted by the remedies directed, or that, at least, she would decide, on cool reflection, to bear the ills she had, for I had not much hope that she would survive an operation, and still less inclination to perform it. I was therefore, not a little startled, about the middle of March last, by the tall, lean figure of Mrs. McLaughlin stalking into my office, with undiminished abdominal protuberance.

She had made her second journey, as her first, to the city, under the friendly cover of a neighbor's wagon, and this time seemed resolved to be rid of her burden. Seeing no help for it, I determined to make a virtue of necessity, and promised to do for her all that she desired.

Mrs. McLaughlin, at her second appearance, was not so well as at the first; the tumour had not perhaps sensibly increased in size, but she had emaciated perceptibly, and there was a frequency and quickness of pulse that betokened hectic irritation of her system. Her appetite had become poor; her bowels were irregular, and the tongue red and sore. It seemed to be proper to get her system into a better state before resorting to an operation, so likely to test its powers of endurance; and accordingly she got mild alterative and aperient medicines every day till the secretions became healthy, the tongue more natural, and the pulse less accelerated, when, on the 29th of March, 1848, a council was convened, consisting of Professor Gross, Drs. T. L. Caldwell, Bayless, and Colescott, to decide upon the propriety of an operation. It was judged to be advisable, at that time, merely to puncture the tumour, where fluctuation was most evident, with the view of diminishing its size, and allowing further time for the system to get into a better condition. I accordingly punctured it with a trochar, in the linea alba, about three inches below the umbilicus, and discharged *twelve pints*, by measure, of a whitish, ropy, albuminous-looking fluid. When the fluid ceased to flow, the abdomen was flaccid to the left of the linea alba, and a short distance to the right; but in the right iliac region, reaching up to the ribs of the same side, it still felt hard and unaltered in shape. No unpleasant symptoms followed the operation, and the puncture soon healed.

April 6th, 1848. With the concurrence and valuable assistance of Professor Gross, and Drs. Bayless, Colescott, W. B. Caldwell, and T. G. Richardson, and in the presence of several students, I performed the operation of extirpating the diseased ovarium. The patient being placed upon a table, on her back, her feet resting upon a chair, and the chloroform having been administered, an incision was made through the integuments, over the linea alba, from the umbilicus to the pubes. The fasciæ and peritoneum were then divided, by several strokes of the scalpel, at the upper part of the incision, and a finger introduced to guide the probe-pointed bistoury, with which an incision was made through these tissues corresponding to that of the integuments. The tumor was now brought to view, and found adherent, though not very firmly, to the omentum and parietes of the abdomen. To break up these adhesions, obstetrician-like, I passed my hand into the abdominal cavity and around the tumor, and had no difficulty in accomplishing the object. It was soon ascertained that the tumor was too large to be pushed through the opening that had been made, but rather than extend this, I punctured two of the cysts, and discharged a quantity of fluid, resembling that which had been drawn off by tapping a week previously,—the quantity could not be estimated, as it was suffered to run upon the table, whence it flowed across the room. By getting my hand posterior to it, I now drew the tumor through the incision, and supported it while Professor Gross secured its pedicle, by a strong ligature, passed through the broad ligament, and tied round the Fallopian tube and ligament of the ovary, and then severed its connections by cutting across the broad ligament near the inferior part of the diseased ovary. Finding that the vessels of the outer extremity of the broad ligament bled considerably, these were secured by another ligature which included the whole of its margin.

The tumor being removed, the ovarian fluid which had been effused, in considerable quantity, into the abdominal cavity, and likewise the blood, were carefully sponged out, and the incision was closed, as usual, by the interrupted suture, adhesive strips, compresses, and bandage, the ligatures having been brought out at the inferior part of the incision. The operation was commenced at 1 o'clock, P. M., and the patient was put to bed in less than an hour, having borne it remarkably well; in fact, as she declared, very little pain, being under the influence of the chloroform.

Five o'clock, P. M., same day. The patient is comfortable; pulse sixty-eight, and volume good; she has vomited once, and complains still of occasional nausea. Gave her thirty drops of laudanum, which is to be repeated in an hour, if not relieved. Alarmed by discharge from the wound, immediately after vomiting, and supposing that there was internal rupture, she sent for me at 10 o'clock at night; although she seemed very much as at the evening visit; to satisfy her the dressings were removed, and it was discovered that there had been a free escape of bloody serum from the inferior angle of the incision.

April 7th—Morning. Found my patient comfortable; no pain or sickness, or tenderness of the abdomen; but she had slept little or none; had not urinated, and said she could not in the recumbent position to which she was strictly confined. Catheter. Ordered to keep quiet, and to have no nourishment but rice, rice-water, or weak tea and toast.

Evening. Slight febrile excitement; pulse seventy-two; skin warm; had slept some, and had passed urine.

Eighth—Morning. Had slept some; was comfortable, complaining only of weariness of confinement to back; had urinated; appetite; pulse sixty-eight, and good.

Evening. Pulse as in the morning, and nothing worth noticing had occurred.

Ninth—Morning. Had slept; cheerful; pulse sixty-eight; no pain; tongue slightly furred; bowels not having been open since the operation, attempted to relieve them by a large warm-water injection, but very little fæces were brought away by it.

Evening. Says she had some pain in the bowels, during the day, tongue not more coated than in the morning; a little fulness in the epigastrium; directed a dose of pills containing blue mass, rhubarb, aloes, and soap.

Tenth—Morning. The pills having failed to operate, used the syringe again, but without effect; no pain or swelling of abdomen; rested better last night than since the operation; pulse sixty-eight.

Evening. Bowels not having acted, ordered tablespoonful of castor oil, to be repeated, if necessary.

Eleventh—Morning. The oil had purged freely, several times, in the course of the night; no complaint.

Evening. Nothing to note.

Henceforward a diary would not be interesting; suffice it to say, that no unfavorable symptoms occurred, at any time; her appetite gradually became good; the bowels acted naturally, or at most she took a little oil once or twice. The wound was examined on the 16th of the month, but finding that it was not yet firmly united, the sutures were not cut, nor were the ligatures pulled. On the 19th, there being some suppuration about the threads, the sutures were cut and removed, and the adhesive strips, &c., replaced ; the ligatures were not touched. On the 25th, one of the ligatures yielded to gentle traction and was removed, but the other, although gently pulled at every dressing, did not yield till the 7th May, at which time the wound was firmly closed, except the small opening through which the ligatures were brought out. The next day, she crossed the river to New Albany, where she had relations, to look out for the same friendly kind of conveyance home that had brought her to the city. I have only heard since that she got a wagon to her notion, and there is no reason to doubt that Mrs. McLaughlin is at this time as notable a housewife as in days of yore.

NOTE.—The tumor, notwithstanding the tapping and the puncture of two of its cysts, at the time of the operation, weighed nine and a quarter pounds. It is composed of many cysts, of various sizes, having strong, thick walls, with smooth, polished linings, and filled, as many of them as were opened for inspection, with the same ropy, white fluid, as that discharged by the trochar. It is preserved in alcohol.—*Western Journ. of Med. and Surg.*

